# Clinician Conceptualization of the Benefits of Treatments for Individual Patients

**DOI:** 10.1001/jamanetworkopen.2021.19747

**Published:** 2021-07-21

**Authors:** Daniel J. Morgan, Lisa Pineles, Jill Owczarzak, Larry Magder, Laura Scherer, Jessica P. Brown, Chris Pfeiffer, Chris Terndrup, Luci Leykum, David Feldstein, Andrew Foy, Deborah Stevens, Christina Koch, Max Masnick, Scott Weisenberg, Deborah Korenstein

**Affiliations:** 1 Department of Epidemiology and Public Health, University of Maryland School of Medicine, Baltimore; 2 VA Maryland Healthcare System, Baltimore; 3 Department of Health, Behavior and Society, Johns Hopkins Bloomberg School of Public Health, Baltimore, Maryland; 4 Adult and Child Consortium of Health Outcomes Research and Delivery Science (ACCORDS), University of Colorado School of Medicine, Aurora; 5 Division of Cardiology, University of Colorado School of Medicine, Aurora; 6 Center of Innovation for Veteran-Centered and Value-Driven Care, VA Denver, Denver, Colorado; 7 Division of General Internal Medicine and Geriatrics, Department of Medicine, Oregon Health and Science University, Portland; 8 Department of Medicine, Dell Medical School, the University of Texas at Austin; 9 South Texas Veterans Health Care System, San Antonio; 10 Department of Medicine, University of Wisconsin School of Medicine and Public Health, Madison; 11 Department of Medicine, Penn State College of Medicine, Hershey, Pennsylvania; 12 Department of Public Health Sciences, Penn State College of Medicine, Hershey, Pennsylvania; 13 Department of Medicine, University of Maryland School of Medicine, Baltimore; 14 Genomic Medicine Institute, Geisinger, Danville, Pennsylvania; 15 New York University Grossman School of Medicine, New York; 16 Memorial Sloan Kettering Cancer Center, New York, New York

## Abstract

**Importance:**

Knowing the expected effect of treatment on an individual patient is essential for patient care.

**Objective:**

To explore clinicians’ conceptualizations of the chance that treatments will decrease the risk of disease outcomes.

**Design, Setting, and Participants:**

This survey study of attending and resident physicians, nurse practitioners, and physician assistants was conducted in outpatient clinical settings in 8 US states from June 2018 to November 2019. The survey was an in-person, paper, 26-item survey in which clinicians were asked to estimate the probability of adverse disease outcomes and expected effects of therapies for diseases common in primary care.

**Main Outcomes and Measures:**

Estimated chance that treatments would benefit an individual patient.

**Results:**

Of 723 clinicians, 585 (81%) responded, and 542 completed all the questions necessary for analysis, with a median (interquartile range [IQR]) age of 32 (29-44) years, 287 (53%) women, and 294 (54%) White participants. Clinicians consistently overestimated the chance that treatments would benefit an individual patient. The median (IQR) estimated chance that warfarin would prevent a stroke in the next year was 50% (5%-80%) compared with scientific evidence, which indicates an absolute risk reduction (ARR) of 0.2% to 1.0% based on a relative risk reduction (RRR) of 39% to 50%. The median (IQR) estimated chance that antihypertensive therapy would prevent a cardiovascular event within 5 years was 30% (10%-70%) vs evidence of an ARR of 0% to 3% based on an RRR of 0% to 28%. The median (IQR) estimated chance that bisphosphonate therapy would prevent a hip fracture in the next 5 years was 40% (10%-60%) vs evidence of ARR of 0.1% to 0.4% based on an RRR of 20% to 40%. The median (IQR) estimated chance that moderate-intensity statin therapy would prevent a cardiovascular event in the next 5 years was 20% (IQR 5%-50%) vs evidence of an ARR of 0.3% to 2% based on an RRR of 19% to 33%. Estimates of the chance that a treatment would prevent an adverse outcome exceeded estimates of the absolute chance of that outcome for 60% to 70% of clinicians. Clinicians whose overestimations were greater were more likely to report using that treatment for patients in their practice (eg, use of warfarin: correlation coefficient, 0.46; 95% CI, 0.40-0.53; *P* < .001).

**Conclusions and Relevance:**

In this survey study, clinicians significantly overestimated the benefits of treatment to individual patients. Clinicians with greater overestimates were more likely to report using treatments in actual patients.

## Key Points

**Question:**

How do clinicians conceptualize the benefits of treatments for common diseases?

**Findings:**

In this survey study of 542 clinicians, most respondents significantly overestimated the benefits of common therapies. Clinicians who conceptualized a greater chance of benefits of therapy were more likely to treat similar patients in their practice.

**Meaning:**

In this study, most clinicians were not well prepared to estimate individual patient chance of benefit, suggesting that an improved understanding of the effects of treatments could lead to more precise use of therapies and better patient outcomes.

## Teaser

This survey study explores clinicians’ conceptualizations of the chance that treatments will decrease the risk of disease outcomes for individual hypothetical patients.

## Introduction

Developing appropriate care plans requires that clinicians and patients have an understanding of the safety and effectiveness of medical treatments.^1^ Scientific evidence of the effects of treatment are generally determined by randomized clinical trials, which compare the outcomes of a disease in a group given therapy vs a control group. These therapeutic effects can be expressed in terms of the absolute or relative reduction in risk of an undesirable outcome.

Clinicians considering prescribing or patients considering taking a therapy need to know how likely a therapy is to benefit patients individually. This can be determined by estimating the risk of an adverse outcome of disease for an individual patient and applying the expected relative risk reduction (RRR) from the therapy to identify the absolute decrement in risk for that patient, known as absolute risk reduction (ARR). While RRR is a property of a therapy across risk groups, ARR best reflects the potential benefit to an individual patient.^2,3,4,5,6,7,8^ Absolute numbers are the recommended metric for physician and patient quantification of benefits of therapies.^4,9^ The chance of benefit from a therapy, along with chance of harms, can then be used by clinicians and patients when considering treatment decisions.^10,11^ Recent public misstatements regarding the potential impact of plasma therapy for COVID-19 illustrates how conflation of RRR and ARR can be misleading. Interpreting an RRR of 35%, the Food and Drug Administration (FDA) commissioner stated that “35 out of 100 patients ‘would have been saved because of the administration of plasma.’”^12^ However, the ARR was actually 3%.^12^ Describing treatment benefits in terms of RRR provides a much larger number than ARR and is associated with patients choosing therapy.^5^

Past studies have found that clinicians overestimated the benefits of treatments. However, these studies were limited by response bias, small sample size, or a focus on statistical language or technical calculations rather than realistic clinical cases.^13,14^ We performed a multicenter survey of clinicians in primary care practice to explore clinician estimations and communication of the expected effects of standard therapy on risk of adverse outcomes for common clinical scenarios.

## Methods

### Survey

We developed a survey to assess clinician estimation of the expected effects of common treatments on disease outcomes and correlate estimates with reported practice. We chose scenarios in which guidelines do not give definitive recommendations and shared decision-making with patients is often recommended. The survey also included other aspects of risk perceptions, which have been presented elsewhere.^15^

A draft survey was developed by primary investigators based in part on previous surveys of risk understanding.^16,17,18^ This survey was reviewed by an expert panel of clinicians with different areas of expertise, practicing in community and academic settings. The survey was further revised by the expert panel during an in-person meeting and 2 conference calls. The survey was piloted with 10 clinicians for comprehension and interpretation of questions, and minor language adjustments were made.

Institutional review board approval was obtained from coordinating sites in Baltimore, Maryland; San Antonio, Texas; and Portland, Oregon. The institutional review boards approved this study with a waiver of informed consent because data were deidentified. The survey was performed in accordance with the American Association for Public Opinion Research (AAPOR) Standard Definitions report.^19^

### Clinician Risk Understanding

The survey assessed risk estimation for common medical therapies used by primary care clinicians in routine care in situations in which treatment is informed by understanding of the probability of benefit. This was similar to previous small surveys.^16,17,18^ Individual testing questions related to treatment of atrial fibrillation, hypertension, osteoporosis, and hypercholesterolemia (eAppendix 1 in the Supplement).

Clinicians were presented with a clinical scenario and asked to estimate the probability of adverse outcome of disease and the effect of treatment. Each scenario was created to represent a general situation but included essential details needed to estimate risk for patients (eg, age and risk factors). Responses to estimates of treatment effect on a disease outcome were compared with scientific estimates using both ARR and RRR. Additional questions were designed to assess whether errors in understanding treatment effects were associated with inaccurate estimates of the risk of adverse outcomes of the disease or a poor understanding of the reduction from treatment.

To assess the accuracy of participant responses, we identified evidence-based estimates of the risk of disease outcome and the effect of medical therapy using a hierarchical method. We first reviewed high-quality recent systematic reviews and meta-analyses and widely accepted risk calculators. If only older systematic reviews and meta-analyses were available, with newer high-impact studies after publication, we considered data from both (attempting to understand the most accurate numbers for current technology and practice). If no systematic reviews and meta-analyses were available, we used data from studies commonly cited in recent guidelines, creating weighted averages by consensus. The expert panel of physicians overseeing the study was presented with best evidence identified, had a comment and question period, and determined consensus evidence-based answers that were used in the analysis (eAppendix 2 in the Supplement).

### Enrollment Procedure

Each coordinating site had a primary investigator (PI) and coordinator. After obtaining institutional review board approval, we contacted the leadership of group practices or residency programs with information related to the study. Coordinators and PIs then approached practices locally and in geographically adjacent states. Investigators sought permission to give a short presentation or email introduction describing the study during a group practice meeting. Individual clinicians were then approached by a coordinator and/or PI to request participation. Lists of clinicians practicing in that clinic were reviewed. Clinicians were eligible for enrollment if they were a physician (MD or equivalent), physician assistant (PA), or nurse practitioner (NP) and cared for patients in a participating outpatient clinical area during the study dates. Clinicians were excluded if they had not seen patients in the past month.

The survey was administered in paper format. The coordinator generally remained at the clinic, office, or meeting location until the clinician had completed the survey. If clinicians requested to complete the survey later, they were provided with an addressed, stamped envelope and could return the survey by mail, email, or by leaving it in the clinic for pick-up. Respondents were provided with a US $50 gift card for completion, if not limited by terms of employment.

Clinicians who initially agreed to participate but did not return the survey within 2 weeks were contacted by study staff via email and/or in person as many as 5 times during a period of up to 3 months. Clinicians who did not complete the survey after these subsequent contacts were considered nonparticipants. Clinicians who declined to participate either at initial enrollment or after reminders were asked to provide a reason for not participating from a standardized list to assess for selection bias.

### Statistical Analysis

Survey responses were entered into a REDCap database with double data entry. Data were analyzed with R version 4.0.0 (R Project for Statistical Computing) for the creation of density plots. SAS version 9.4 (SAS Institute) was used for descriptive statistics and all other statistical analyses. Comparison of those who completed all key survey questions and those who did not was done with a χ^2^ test. To test the correlation between estimates of benefits and clinical decisions, we used *t* test based on a transformation of the correlation coefficient, as implemented in SAS. A sample size of 500 was planned based on desire for generalizable results across enrollment sites. Statistical significance was set at *P* < .05, and all tests were 2-tailed.

## Results

### Participant Demographic Characteristics

The survey was offered to 723 primary care physicians, NPs, and PAs practicing in Delaware, Maryland, Oregon, Pennsylvania, Texas, Virginia, Washington, and the District of Columbia (Table 1). The overall response rate was 81% (585 of 723). Of the 585 clinicians who returned the survey, we excluded 43 who did not complete all questions necessary for analysis, leaving a final sample of 542 (Table 1).

**Table 1.** Recruitment of Participants by Study Site and Reasons for Lack of Participation

Participation status	Participants, No. (%)			
	Maryland and mid-Atlantic states (n = 390)	Oregon and Washington (n = 150)	Texas (n = 183)	All sites (N = 723)
No response	41 (10)	0	16 (9)	57 (8)
Refusals^a^				
Total	10 (2)	3 (2)	3 (1)	16 (2)
Not interested	2 (1)	0	2 (1)	4 (1)
Too busy or bad timing	6 (2)	2 (1)	2 (1)	10 (1)
Too difficult	2 (1)	1 (1)	0	3 (<1)
Other	3 (1)	0	0	3 (<1)
Agreed to participate	339 (87)	147 (98)	164 (90)	650 (90)
Agreed but did not complete survey	27 (7)	23 (15)	15 (8)	65 (9)
Total surveys received	312 (80)	124 (82)	149 (81)	585 (81)
Failed to complete ≥1 questions required for final analysis	16 (4)	11 (7)	16 (9)	43 (6)
Final sample for analysis, No.	296	113	133	542

^a^ May list more than 1 reason for refusing to complete the survey.

Overall, 480 respondents (89%) were physicians with MD or DO degrees; 282 (52%) were in residency; 194 (52%) identified as White, 138 (25%) as Asian, 26 (6.6%) as Black, and 44 (8.1%) as Hispanic. The median (interquartile range) age was 32 (29-44) years, and 290 respondents (54%) were women (Table 2). The survey required a median (IQR) of 20 (15-25) minutes to complete.

**Table 2.** Demographic Characteristics and Other Practice Factors Among Enrolled Health Care Workers

Characteristic	Participants, No. (%)
Degree	
MD or equivalent	480 (89)
NP	48 (9)
PA	14 (3)
Race	
White	294 (54)
Black	36 (7)
Asian	138 (26)
Hispanic/Latino	44 (8)
≥1 race	18 (3)
Other or missing^a^	10 (2)
Female gender	290 (54)
Male gender	252 (56)
Age, median (IQR), y	32 (29-44)
Medical, nursing, or PA school	
International	103 (19)
DO	20 (4)
Current resident	282 (52.0)
Type of residency	
Internal medicine	326 (60.1)
Family medicine	141 (26.0)
Other or NA	75 (13.8)
Type of practice^b^	
Academic	331 (54.4)
Rural	7 (1.1)
Suburban	58 (9.5)
Urban	81 (13.3)
VA	126 (20.7)
Ever sued for malpractice	28 (5.3)
Other graduate degree	114 (21.6)
Time practice, median (IQR), y	
All respondents	3 (1-11)
Residents	2 (1-3)
Nonresidents	11 (5-21)

Abbreviations: IQR, interquartile range; NA, not applicable; NP, nurse practitioner; PA, physician assistant; VA, Veterans Affairs.

^a^ Other race included those identifying as American Indian/Alaskan Native or Native Hawaiian/Pacific Islander.

^b^ A total of 609 types of practices were reported for 542 participants, as respondents could select more than 1 type.

We were unable to collect any information to characterize nonresponders. We compared the 43 respondents who did not complete all necessary questions with the final cohort of 542 clinicians with complete responses. We found those not completing the survey, compared with those who did complete the survey, were more likely to be women (31 [72%] vs 290 [54%]; *P* = .02), to be residents (13 [30%] vs 282 [52%]; *P* = .006), and to be NPs or PAs (12 [29%] vs 62 [11%]; *P* = .001).

### Estimates of Adverse Outcomes of Disease and Effects of Treatment

**Figure 1.**
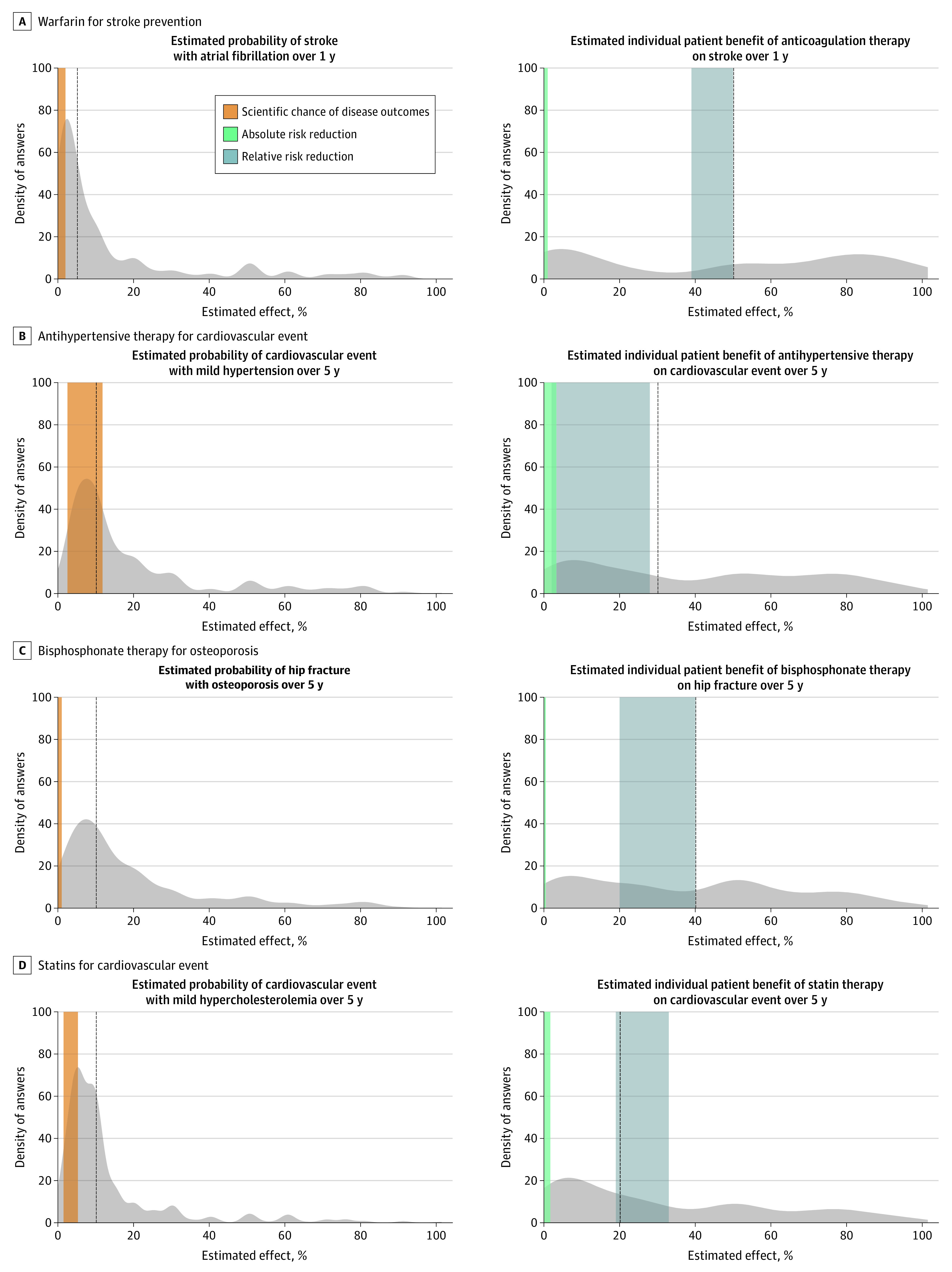
Clinician Estimates of Likelihood of Disease Outcomes and Benefits of Treatment Gray shaded areas indicate the frequency distribution of responses; the dotted vertical line identifies the median answer.

Clinician estimates of the probability of benefit from medical therapy were consistently higher than scientific evidence for ARR or RRR (Figure 1). Estimates did not significantly differ between trainees and clinicians in practice (eTable in the Supplement).

In the atrial fibrillation clinical scenario, the median (IQR) estimated probability of stroke with atrial fibrillation without treatment was 5% (2%-15%), while evidence supported a rate of 0.4% to 2%. The median (IQR) estimated chance that warfarin would prevent a stroke in that patient in the next year was 50% (5%-80%) compared with evidence revealing an ARR of 0.2% to 1% from an RRR of 39% to 50% for stroke prevention. Clinician estimates of benefits from therapy exceeded the total estimated chance of an adverse disease outcome in 379 responses (70%). The estimated benefits of therapy exceeded the upper limit of RRR for 235 clinicians (43%).

In the mild hypertension scenario, the median (IQR) estimated probability of a cardiovascular event without treatment was 10% (6%-20%), while evidence supported a rate of 3% to 12%. The median (IQR) estimated chance that antihypertensive therapy would prevent a cardiovascular event in that patient within 5 years was 30% (10%-70%), while evidence supported an ARR of 0% to 3.3% from an RRR of 0% to 28%. Clinician estimates of benefits from therapy exceeded the total estimated chance of an adverse disease outcome for 340 responses (63%). The estimated benefits of therapy exceeded the upper limit of RRR for 294 clinicians (54%).

In the osteoporosis scenario, the median (IQR) estimated probability of hip fracture without treatment was 10% (5%-25%), while evidence supported a rate of 0.3% to 1%. The median (IQR) estimated chance that bisphosphonate therapy would prevent a hip fracture in that patient in the next 5 years was 40% (10%-60%) compared with evidence expressed most accurately as an ARR of 0.1% to 0.4% from an RRR of 20% to 40%. Clinician estimates of benefits from therapy exceeded the total estimated chance of an adverse disease outcome for 343 responses (63%). The estimated benefits of therapy exceeded the upper limit of the RRR for 248 clinicians (46%).

In the mild hyperlipidemia scenario, the median (IQR) estimated probability of cardiovascular event without treatment was 10% (5%-15%), while evidence supported a rate of 2% to 5%. The median (IQR) estimated chance that moderate-intensity statin therapy would prevent a cardiovascular event in that patient in the next 5 years was 20% (5%-50%) vs evidence of an ARR of 0.3% to 2% from an RRR of 19% to 33%. Clinician estimates of benefits from therapy exceeded the total estimated chance of an adverse disease outcome for 323 responses (60%). The estimated benefits of therapy exceeded the upper limit of the RRR for 204 clinicians (38%).

A hypothetical scenario addressing test interpretation revealed better understanding than with clinically grounded questions. For the question, “Consider a condition in which 3% of patients will develop a bad outcome within 5 years. Treatment has a relative risk reduction of 33%. If 100 patients with this condition are treated for 5 years, how many patients will have a bad outcome prevented by treatment?” the median (IQR) response was 2 (1-20) of 100 patients. (The correct answer was 1 of 100 patients.)

### Association Between Estimates of Treatment Effects and Recommending Treatment to Patients

**Figure 2.**
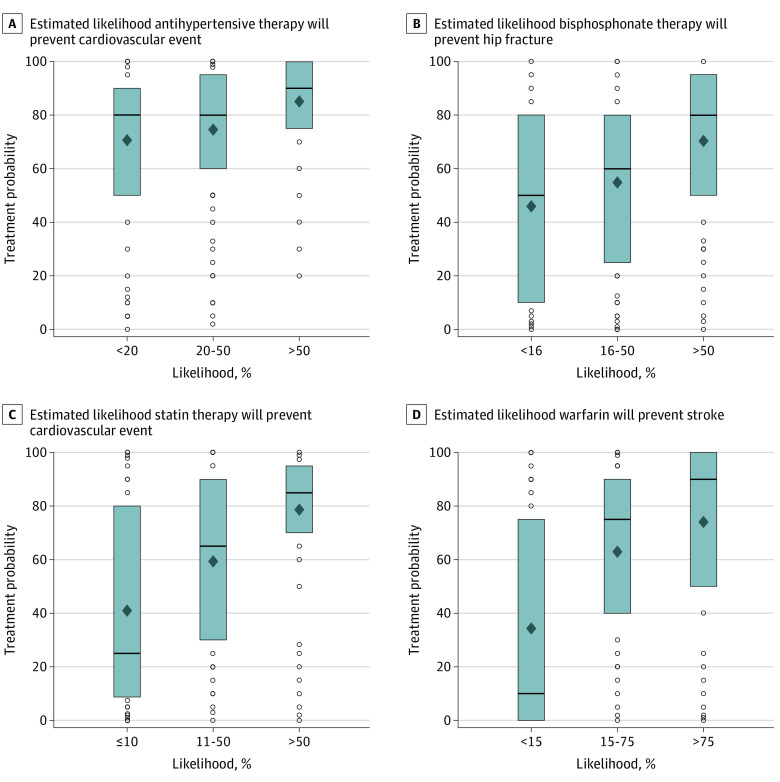
Association Between Clinician Estimate of Benefit and Use of Treatment in Real Life Patients The boxes represent the middle 50% of data, with the lines representing the median. Diamonds indicate means, and dots indicate values outside the interquartile range.

To evaluate whether estimates of benefits of medical therapy correlated with clinical practice decisions, we compared 2 answers from each respondent: (1) “What will you tell [a specific patient] is the chance that [a treatment] will prevent them from having [a specific disease outcome]?” and (2) “In your practice, in what proportion of patients with similar risk do you recommend treatment?” We found a moderate positive correlation for each treatment scenario between estimates of the degree to which treatment prevented a disease outcome and how often they recommended such treatment in their real-life patients (eg, use of warfarin: correlation coefficient, 0.46; 95% CI, 0.40-0.53; *P* < .001) (Figure 2). For the scenario involving atrial fibrillation, clinicians answering near the ARR range of 1% chance of preventing a stroke in this patient recommended treatment in approximately 35% of similar patients in their practice, while the median clinician estimated a 50% chance of benefit and reported treating approximately 60% of similar patients in their practice. Similar effects were seen with other scenarios.

## Discussion

In scenarios routinely encountered in practice, clinicians significantly overestimated the benefits of medical therapy. When asked, “What will you tell [a specific patient] is the chance that [a treatment] will prevent them from having [a specific disease outcome]?” clinicians rarely responded with estimates in the range of the ARR, as recommended for clinical decision-making. Most clinicians estimated individual benefits in the range of RRR, and one-third to one-half estimated benefits beyond the RRR. Clinicians who overestimated benefits of treatment were more likely to report recommending that treatment to similar patients in their own practice.

Few clinicians in our study appeared to use ARR to anchor estimates of individual patient benefit. ARR is the best understood and most helpful metric of impact of therapy for an individual patient,^20,21^ but it is often small compared with RRR. Not surprisingly, RRR is often preferentially reported in the literature for research with small effect sizes or by the pharmaceutical industry, practices that have been criticized.^22,23,24^ Clinicians do not receive extensive training or a consistent method to consider potential benefits and harms of therapy for individual patients, and many clinicians learn treatment approaches from yes/no–type guideline recommendations or consider benefits based on clinical experience, physiological thinking, or other theoretical models.^25,26,27^

We are uncertain whether clinicians conceptualize patient benefit using evidence-based medicine metrics. It is clear clinicians rarely conceptualize benefit in terms of ARR. Their estimates suggest conceptualization of individual benefit to a patient in general terms closer to RRR and not ARR. Most respondents estimated the chance that the patient would benefit from a treatment to be a greater than the total chance that the same patient would have a negative outcome from disease. This response is only logically consistent if outcomes are conceptualized in absolute chance and benefits in relative terms. For example, in the atrial fibrillation scenario, the average clinician estimated that for a specific patient, Mr Miller, the likelihood of stroke was 5% and the chance that warfarin therapy would prevent Mr Miller from having a stroke was 50%. This is despite the survey question posing, “What would you tell Mr. Miller is the chance that warfarin will prevent *him* from having a stroke in the next year?” For the questions in our survey, the 20% to 50% estimated chance of benefit from common treatments to a patient would dramatically inflate expected benefit compared with the recommended ARR, which ranged across examples from 1% to 3%.

Notably, in our study, overestimates of treatment benefit often went beyond RRR. For each question, one-third to one-half of estimates of benefit were beyond the upper end of the RRR. This would imply that many clinicians either grossly overestimated the RRR for common treatments or that they did not conceptualize patient benefit from therapy in the evidence-based medicine approach of using language of chance or probability.

In contrast to the clinical scenarios presented, in 1 hypothetical question, clinicians correctly calculated ARR from RRR. The ability to technically calculate ARR while reverting to the much higher estimates of RRR to understand commonly prescribed treatments implies evidence-based principles of treatment may be understood theoretically but are not understood or applied to clinical care.^28^ The observed overestimates of benefit are also consistent with cognitive biases that have been described on other contexts, including base rate neglect, anchoring bias, and confirmation bias,^29,30,31^ as well as the observation that humans often exaggerate small risks.^32^

Our survey presented clinical scenarios that are common in primary care. For the patients described, guidelines would generally not recommend treatment for atrial fibrillation^33^ or osteoporosis^34^; treatment of hypertension and hypercholesterolemia could possibly be recommended if unreported risk factors were present.^35,36^ We found that in these patient scenarios, physicians who overestimated treatment benefit were more likely to report treating similar patients in their own practice. This suggests that overestimates of treatment benefit may directly affect clinical decisions, potentially exposing patients to therapies for which potential harms outweigh benefits. This matches studies finding frequent overuse of treatment for low-risk patients, such as in atrial fibrillation.^37^

Clinician overestimates of treatment benefits would severely limit shared decision-making. Shared decision-making is broadly recommended^38,39^ and is often invoked as a key component for improving care.^40,41,42^ When clinicians engage in shared decision-making, there is a general consensus that patients should be presented with harms and benefits, using absolute and not relative numbers.^1,2,11,22,43^ Training in shared decision-making has focused on communication skills but may also require training on probability of benefit from treatment.^44^ While the optimal approach to improve these skills has not been described, educational approaches, risk calculators, decision aids, and behavioral nudges may be helpful.

### Limitations

This study has limitations, including that the small fraction of respondents who did not complete the survey were more likely to be women, NPs, or PAs. However, the overall response rate was high. While validity was extensively assessed via a multidisciplinary expert panel, reliability of our novel survey was not assessed. We requested estimates of benefits and interpreted conceptualization from answers provided. This gave more concrete answers but did not fully assess how treatment benefit was conceptualized by individual clinicians and may not capture the totality of communication in a clinical encounter.

## Conclusions

In this study, clinicians performing primary care significantly overestimated the chance that common therapies would benefit individual patients. Clinicians with the largest overestimates were also likely to prescribe these treatments in patients in their practice. Widespread overestimates of the benefits of therapy likely contribute to overtreatment in actual patients. Improved understanding of the effects of treatments is essential for precise use of therapies and better patient outcomes.

## Notes

Supplement. **eAppendix 1. **Survey Questions **eAppendix 2.** Determination of Best Answers for Treatment Questions eReferences. **eTable.** Comparison of Resident Physicians vs Primary Care Clinicians for Estimates of Probability of Disease and Individual Benefit With Treatment
