# Supplementary material for: Clinician Conceptualization of the Benefits of Treatments for Individual Patients
Source: JAMA Netw Open. 2021 Jul 21;4(7):e2119747. doi: 10.1001/jamanetworkopen.2021.19747 (PMC8295738; doi:10.1001/jamanetworkopen.2021.19747)
Supplement: Supplement. — eAppendix 1. Survey Questions eAppendix 2. Determination of Best Answers for Treatment Questions eReferences. eTable. Comparison of Resident Physicians vs Primary Care Clinicians for Estimates of Probability of Disease and Individual Benefit With Treatment [file jamanetwopen-e2119747-s001.pdf]

Supplemental Online Content

Morgan DJ, Pineles L, Owczarzak J, et al. Clinician conceptualization of the benefits of treatments for individual patients. *JAMA Netw Open.* 2021;4(7):e2119747. doi:10.1001/jamanetworkopen.2021.19747

eAppendix 1. Survey Questions

eAppendix 2. Determination of Best Answers for Treatment Questions

eReferences.

eTable. Comparison of Resident Physicians vs Primary Care Clinicians for Estimates of Probability of Disease and Individual Benefit With Treatment

This supplemental material has been provided by the authors to give readers additional information about their work.

© 2021 Morgan DJ et al. *JAMA Network Open.*

eAppendix 1. Survey Questions

1. **Consider Mr. Miller, a 62-year-old man with well-controlled hypertension on aspirin with new atrial fibrillation. He has no particular preference for treatment and wants your advice.**

What is Mr. Miller's risk for stroke in the next year with no change in therapy? _______%

What would you tell Mr. Miller is the chance that warfarin will prevent him from having a stroke in the next year? _______%

IN YOUR PRACTICE, in what proportion of patients with atrial fibrillation and similar risk to Mr. Miller do you recommend anticoagulation? _______%

2. **Consider Mr. Davis, a 60-year-old man with persistent mild hypertension (SBP 145/92) without preexisting cardiovascular disease. He is adherent to lifestyle interventions and has no particular preference for treatment and wants your advice.**

How likely is Mr. Davis to have a cardiovascular event within 5 years with no antihypertensive therapy? _______%

What would you tell Mr. Davis is the chance that antihypertensive therapy will prevent him from having a cardiovascular event within 5 years? _______%

IN YOUR PRACTICE, in what proportion of patients with mild hypertension with similar risk to Mr. Davis do you recommend antihypertensive therapy? _______%

3. **Consider Mrs. Wilson, a 60-year-old Caucasian woman with BMI 30 and osteoporosis without fracture. She is active and has adequate dietary calcium and vitamin D. She has no particular preference for treatment and wants your advice.**

How likely is Mrs. Wilson to have a hip fracture in the next 5 years with no additional treatment? _______%

What would you tell Mrs. Wilson is the chance that bisphosphonate therapy (e.g. alendronate) will prevent her from having a hip fracture in the next 5 years? _______%

IN YOUR PRACTICE, in what proportion of patients with osteoporosis and similar risk to Mrs. Wilson do you recommend bisphosphonate therapy? _______%

4. **Consider Mr. Brown, a healthy 52-year-old man who is found to have a screening LDL of 150 and HDL of 40. He has tried lifestyle interventions, has no particular preference for treatment and wants your advice.**

How likely is Mr. Brown to have a cardiovascular event within 5 years with no additional treatment? _______%

What would you tell Mr. Brown is the chance that moderate-intensity statin therapy will prevent him from having a cardiovascular event in the next 5 years? _______%

IN YOUR PRACTICE, in what proportion of patients with hypercholesterolemia and similar risk to Mr. Brown do you recommend statins? _______%

5. **Consider a condition in which 3% of patients will develop a bad outcome within 5 years and a treatment has a relative risk reduction of 33%.**

If 100 patients with this condition are treated for 5 years, how many patients will have a bad outcome prevented by treatment? _______%

© 2021 Morgan DJ et al. *JAMA Network Open.*

eAppendix 2. Determination of Best Answers for Treatment Questions

To identify the best evidence-based information from the literature regarding probability of a disease outcome and impact of treatment on individual patients[1], we used a hierarchical method.

1. Data was first sought from high-quality recent systematic reviews, meta-analyses, and/or guidelines.
2. If only older systematic reviews, meta-analyses, and/or guidelines were available with newer high-impact studies after publication, we considered data from both (attempting to understand most accurate numbers for current technology/practice)
3. If no systematic reviews, meta-analyses, and/or guidelines were available, we used data from commonly cited studies based on citations in recent guidelines and creating weighted averages by consensus. The expert panel of physicians overseeing the study was presented with best evidence identified and settled on evidence-based answers presented in results.

Of note, we evaluated recommendations in guidelines around treatment for each individual case. These will not be part of the primary analysis but will likely inform our understanding of results. Each question was written to capture an area with some uncertainty in standard practice—when patient shared decision making would be important.

Quotes are often included in this document. They will mostly be removed from the final appendix but are present to make it easier to evaluate evidence.

© 2021 Morgan DJ et al. *JAMA Network Open.*

Question 1

Consider Mr. Miller, a 62-year-old man with well-controlled hypertension on aspirin with new atrial fibrillation. He has no particular preference for treatment and wants your advice.

a. **What is Mr. Miller's risk for stroke in the next year with no change in therapy?**

To determine the probability of stroke in a patient with atrial fibrillation, we found clinical prediction scores to be the best validated method.
CHA2DS2-VASc is the most accepted score for atrial fibrillation. It gives a point for, hypertension, which would give Mr. Miller a 1.3% risk of stroke in the next year.[2] The CHA2DS2-VASc score is not completely accurate[3] , so we gave a range around the estimate of a relative 50% higher or lower to 0.6%-2.0% absolute risk.

There's controversy about if aspirin decreases risk of stroke in Afib. We modified the risk of stroke lower to capture this uncertainty.[4] So, his risk of stroke could be as low as 0.4%

In summary, risk of stroke in one year for this patient is 0.4% - 2.0%

b. **What would you tell Mr. Miller is the chance that warfarin will prevent him from having a stroke in the next year?**
The benefits of treating atrial fibrillation with anticoagulation to prevent stroke has been reviewed. The most recent review was Steinberg BA, Piccini JP.[5] Which referred back to a systematic review by Hart et al.[6]

This review identified a 39% RRR compared to aspirin, and others have referenced a 50% reduction in comparison to placebo, so the treatment benefit would be 39%-50% relative risk reduction which would translate to a 0.16%-1.0% benefit to Mr. Miller directly.

c. **Do Guidelines recommend treatment?**
Mr. Miller has a CHA2DS2-VASc score of 1. Guidelines from AHA/ACC do NOT recommend anticoagulation for a patient like Mr. Miller.[7]

"For patients with AF and an elevated CHA2DS2-VASc score of 2 or greater in men or 3 or greater in women, oral anticoagulants are recommended."

Question 1 References[2–7]

© 2021 Morgan DJ et al. *JAMA Network Open.*

Question 2

Consider Mr. Davis, a 60-year-old man with persistent mild hypertension (SBP 145/92) without preexisting cardiovascular disease. He is adherent to lifestyle interventions and has no particular preference for treatment and wants your advice.

a. **How likely is Mr. Davis to have a cardiovascular event within 5 years with no antihypertensive therapy?**

To determine the probability of cardiovascular events in a patient with mild[8] hypertension, we found clinical prediction scores to be the best validated method. Cardiovascular risk scores included The ASCVD risk[8] estimator

For risk calculators, cholesterol is a key component. As we didn't discuss cholesterol, we are considering a broad range to include very low cholesterol and very high cholesterol.

Assuming low cholesterol (TC 160, HDL 60, LDL 100) 10-year 7.6% risk
Assuming high cholesterol (TC 300, HDL 40, LDL 180) 10-year risk 17.7%

The proportion of that 10-year risk that is in the first 5 years is likely 1/3-2/3 of the 10-year risk.
So, 1/3 of the lower bound of 10-year risk is 7.6% = 2.53%
And 2/3 of the upper bound of 10-year risk 17.7% = 11.8%

In summary, risk of a cardiovascular event over 5 years for this patient is 2.5-11.8%

b. **What would you tell Mr. Davis is the chance that antihypertensive therapy will prevent him from having a cardiovascular event within 5 years?**
The effect of treatment of his hypertension was identified through a recent systematic review.[9]
These effects are consistent with those identified when this patient is considered using AHA/ACC risk score. ASCVD calculator provides HTN relative risk reduction ~25-26%

Treatment benefit per systematic review: OR 0.72[9] or 28% relative reduction, so absolute benefit would be 28% of 2.53%-(28% of 11.8%). However, this systematic review combined stroke and cardiovascular events, and the effect was non-significant for cardiovascular events alone, so estimates of benefit include 0%. The relative risk reduction was 0-28% and the absolute benefits to Mr. Davis are 0%-3.3%

Do Guidelines recommend treatment?

Guidelines are variable. Cardiology guidelines recommend treatment of patients "Use of BP-lowering medication is recommended for primary prevention of CVD in adults with no history of CVD and with an estimated 10-year ASCVD risk <10% and an SBP of 140 mm Hg or higher or a DBP of 90 mm Hg or higher", whereas primary care guidelines (ACP/AAFP) do not recommend treatment with systolic blood

© 2021 Morgan DJ et al. *JAMA Network Open.*

pressure below 150 without increased cardiovascular risk.[10,11]

ACP/AAFP:
"ACP and AAFP recommend that physicians initiate treatment in adults aged 60 years old and older with persistent systolic blood pressure at or above 150 millimeters of mercury (mm Hg) to achieve a target systolic blood pressure of less than 150 mm Hg to reduce the risk of mortality, stroke, and cardiac events."

"ACP and AAFP recommend that physicians consider initiating or intensifying pharmacological treatment in some adults aged 60 years old and older at high cardiovascular risk, based on individualized assessment, to achieve a target systolic blood pressure of less than 140 mm Hg to reduce the risk of stroke or cardiac events.

Increased cardiovascular risk includes all people with known vascular disease and among others, is defined as most patients with diabetes, individuals with chronic kidney disease with estimated glomerular filtration rate (eGFR) <45 mL/min/per 1.73 m2, metabolic syndrome (abdominal obesity, hypertension, diabetes, and dyslipidemia), and older age."[12]

Question 2 References[9–12]

© 2021 Morgan DJ et al. *JAMA Network Open.*

Question 3

Consider Mrs. Wilson, a 60-year-old Caucasian woman with BMI 30 and osteoporosis without fracture. She is active and has adequate dietary calcium and vitamin D. She has no particular preference for treatment and wants your advice.

a. **How likely is Mrs. Wilson to have a hip fracture in the next 5 years with no additional treatment?**

Chance of hip fracture is less than 1%.

The FRAX prediction model appears to be the best accepted model and estimates a 10-year risk of hip fracture for Mrs. Wilson to be 0.5%, (although 6.5% "major osteoporotic fracture")[13]

When one adjusts weight parameters in the FRAX model, the patient could have up to a 2% 10-year risk of hip fracture. To convert from a 10 year risk, we assumed 1/3 to 2/3 of risk would be in the first 5 years.

Mrs. Wilson therefore has a **0.17%-1% risk of hip fracture in next 5 years**

b. **What would you tell Mrs. Wilson is the chance that bisphosphonate therapy (e.g. alendronate) will prevent her from having a hip fracture in the next 5 years?**

The Crandall & Shekelle systematic review provides a RR 0.6-0.8 for hip fracture if taking bisphosophonates. In other words, relative risk reduction was 20-40%. Given a 0.17%-1.0% risk of hip fracture over 5 years; the benefit would then be 0.1-0.4%[14]

For comparison, the 2014 AIM Crandall & Shekelle article NNT ~80 over 1-3 years for alendronate for vertebral or non-vertebral fractures but provided no NNT for hip fracture alone.[14] Some have discussed there is no definite effect on hip fracture prevention.[15]

Therefore, we conclude that Mrs. Wilson has a 0.17-1% risk of hip fracture in 5 years that will be decreased by 0-0.4% if she takes bisphosphonate therapy.

Do Guidelines recommend treatment?

USPSTF would not recommend screening this patient with DEXA

"The U.S. Preventive Services Task Force (USPSTF) recommends screening for osteoporosis in women age 65 years and older, and in women younger than age 65 years who have been through menopause and are at increased risk for osteoporosis."[16]

© 2021 Morgan DJ et al. *JAMA Network Open.*

For a patient diagnosed with osteoporosis, based on a T-score < -2.5 she would be recommended pharmacologic treatment per Endocrine Society guidelines.[17]

“In the United States, pharmacological therapy is recommended for postmenopausal women with hip or vertebral fractures; those with T-scores of −2.5 or less in the femoral neck, total hip, or lumbar spine”

Question 3 References[13–17]

© 2021 Morgan DJ et al. *JAMA Network Open.*

<u>Question 4</u>

Consider Mr. Brown, a healthy 52-year-old man who is found to have a screening LDL of 150 and HDL of 40. He has tried lifestyle interventions, has no particular preference for treatment and wants your advice.

This is a case of primary prevention for mild hyperlipidemia.

To determine the probability of cardiovascular events in a patient with hyperlipidemia, we found clinical prediction scores to be the best validated method. Similar to question 1, experts noted the ASCVD risk calculator may overestimate risk, so we included a range of risk.

ACC ASCVD Risk Estimator plus[8]

His 10-year risk varies by total cholesterol which is dependent on triglyceride level, which we did not provide. We are therefore providing a range of correct answers:

If total cholesterol = 190, then 10-year risk 4.4% (TGL 0)

If total cholesterol = 300, then 10-year risk 7.9% (TGL 550)

Assuming 1/3 to 2/3 of that risk is in the first 5 years 1.5% to 5.3%

This risk estimate is similar to ACC/AHA guidelines.[18,19]

a. **How likely is Mr. Brown to have a cardiovascular event within 5 years with no additional treatment?**
   1.5%-5.3%

c. **What would you tell Mr. Brown is the chance that moderate-intensity statin therapy will prevent him from having a cardiovascular event in the next 5 years?**
   Relative benefit of statins based on the American Heart Journal: all major CV events RR: 0.74, 95%CI: 0.67-0.81 (19%-33% relative risk reduction)[20] Similarly, the ASCVD Risk estimator uses 25.2% relative risk for all ranges of recommended treatment.

   So, absolute benefit would be 0.3%-1.7%

© 2021 Morgan DJ et al. *JAMA Network Open.*

Do Guidelines recommend treatment?

Guidelines generally don't recommend therapy for this range of risk, but they may if this individual was at the higher end of risk with a high total cholesterol.

ASCVD Risk estimator: Guidelines do not recommend statin therapy for patients with 10-year risk < 5%. However, if risk > 5% it should be considered.

Question 4 References[8,18–20]

© 2021 Morgan DJ et al. *JAMA Network Open.*

eReferences

1. Armstrong KA, Metlay JP. Annals Clinical Decision Making: Translating Population Evidence to Individual Patients. *Annals of Internal Medicine*. 2020;172(9):610-616. doi:10.7326/M19-3496

2. van Walraven C, Hart RG, Connolly S, et al. Effect of age on stroke prevention therapy in patients with atrial fibrillation: the atrial fibrillation investigators. *Stroke*. 2009;40(4):1410-1416. doi:10.1161/STROKEAHA.108.526988

3. Borre ED, Goode A, Raitz G, et al. Predicting Thromboembolic and Bleeding Event Risk in Patients with Non-Valvular Atrial Fibrillation: A Systematic Review. *Thromb Haemost*. 2018;118(12):2171-2187. doi:10.1055/s-0038-1675400

4. January Craig T., Wann L. Samuel, Calkins Hugh, et al. 2019 AHA/ACC/HRS Focused Update of the 2014 AHA/ACC/HRS Guideline for the Management of Patients With Atrial Fibrillation: A Report of the American College of Cardiology/American Heart Association Task Force on Clinical Practice Guidelines and the Heart Rhythm Society in Collaboration With the Society of Thoracic Surgeons. *Circulation*. 2019;140(2):e125-e151. doi:10.1161/CIR.0000000000000665

5. Steinberg BA, Piccini JP. Anticoagulation in atrial fibrillation. *BMJ*. 2014;348. doi:10.1136/bmj.g2116

6. Hart RG, Pearce LA, Aguilar MI. Meta-analysis: antithrombotic therapy to prevent stroke in patients who have nonvalvular atrial fibrillation. *Ann Intern Med*. 2007;146(12):857-867. doi:10.7326/0003-4819-146-12-200706190-00007

7. DeJong J. https://www.chadsvasc.org/. CHA2DS2-VASc/HAS-BLED/EHRA atrial fibrillation risk score calculator.

8. American College of Cardiology. ASCVD Risk Estimator Plus. Accessed July 15, 2020. http://tools.acc.org/ASCVD-Risk-Estimator-Plus/#!/calculate/estimate/

9. Sundström J, Arima H, Jackson R, et al. Effects of blood pressure reduction in mild hypertension: a systematic review and meta-analysis. *Ann Intern Med*. 2015;162(3):184-191. doi:10.7326/M14-0773

10. Whelton Paul K., Carey Robert M., Aronow Wilbert S., et al. 2017 ACC/AHA/AAPA/ABC/ACPM/AGS/APhA/ASH/ASPC/NMA/PCNA Guideline for the Prevention, Detection, Evaluation, and Management of High Blood Pressure in Adults: A Report of the American College of Cardiology/American Heart Association Task Force on Clinical Practice Guidelines. *Hypertension*. 2018;71(6):e13-e115. doi:10.1161/HYP.0000000000000065

11. Whelton PK, Williams B. The 2018 European Society of Cardiology/European Society of Hypertension and 2017 American College of Cardiology/American Heart Association Blood Pressure Guidelines: More Similar Than Different. *JAMA*. 2018;320(17):1749-1750. doi:10.1001/jama.2018.16755

12. Qaseem A, Wilt TJ, Rich R, Humphrey LL, Frost J, Forciea MA. Pharmacologic Treatment of Hypertension in Adults Aged 60 Years or Older to Higher Versus Lower Blood Pressure Targets: A Clinical

© 2021 Morgan DJ et al. *JAMA Network Open.*

Practice Guideline From the American College of Physicians and the American Academy of Family Physicians. *Annals of Internal Medicine*. 2017;166(6):430-437. doi:10.7326/M16-1785

13. Sheffield U of. *FRAX® Fracture Risk Assessment Tool*. https://www.sheffield.ac.uk/FRAX/

14. Crandall CJ, Newberry SJ, Diamant A, et al. Comparative effectiveness of pharmacologic treatments to prevent fractures: an updated systematic review. *Annals of Internal Medicine*. 2014;161(10):711–723. doi:10.7326/M14-0317

15. Crandall CJ. Can We Pave the Pathway to Fracture Prevention? *Annals of Internal Medicine*. 2019;171(1):62–63. doi:10.7326/M19-1112

16. Viswanathan M, Reddy S, Berkman N, et al. Screening to Prevent Osteoporotic Fractures: Updated Evidence Report and Systematic Review for the US Preventive Services Task Force. *JAMA*. 2018;319(24):2532. doi:10.1001/jama.2018.6537

17. *Pharmacological Management of Osteoporosis in Postmenopausal Women: An Endocrine Society* Clinical Practice Guideline \textbar The Journal of Clinical Endocrinology & Metabolism \textbar Oxford Academic*. https://academic.oup.com/jcem/article/104/5/1595/5418884

18. Arnett Donna K., Blumenthal Roger S., Albert Michelle A., et al. 2019 ACC/AHA Guideline on the Primary Prevention of Cardiovascular Disease: Executive Summary: A Report of the American College of Cardiology/American Heart Association Task Force on Clinical Practice Guidelines. *Circulation*. 2019;140(11):e563-e595. doi:10.1161/CIR.0000000000000677

19. Grundy SM, Stone NJ, Bailey AL, et al. 2018 AHA/ACC/AACVPR/AAPA/ABC/ACPM/ADA/AGS/APhA/ASPC/NLA/PCNA Guideline on the Management of Blood Cholesterol: A Report of the American College of Cardiology/American Heart Association Task Force on Clinical Practice Guidelines. *J Am Coll Cardiol*. 2019;73(24):e285-e350. doi:10.1016/j.jacc.2018.11.003

20. Yebyo HG, Aschmann HE, Kaufmann M, Puhan MA. Comparative effectiveness and safety of statins as a class and of specific statins for primary prevention of cardiovascular disease: A systematic review, meta-analysis, and network meta-analysis of randomized trials with 94,283 participants. *American Heart Journal*. 2019;210:18–28. doi:10.1016/j.ahj.2018.12.007

© 2021 Morgan DJ et al. *JAMA Network Open.*

eTable. Comparison of Resident Physicians vs Primary Care Clinicians for Estimates of Probability of Disease and Individual Benefit With Treatment

**Clinical scenario**	**Total population**	**Resident estimate (n=282), % (median, IQR)**	**Primary care clinician estimate (n=260), % (median, IQR)**	**Scientific Evidence, ARR %**
**Atrial Fibrillation**				
Probability of stroke	5% (2-15)	5% (1.3-10)	5% (2-20)	0.4-2%
Estimate of individual benefit with warfarin	50% (5-80)	50% (5-80)	50% (5-80)	0.2-1%
**Hypertension**				
Probability of cardiovascular event	10% (6-20)	10% (5-25)	10% (7-20)	3-12%
Estimate of individual benefit with antihypertensive	30% (10-70)	40% (10-60)	30% (10-70)	0-3%
**Osteoporosis**				
Probability of hip fracture	10% (5-25)	11% (5-30)	10% (5-20)	0.3-1%
Estimate of individual benefit with bisphosphonate therapy	40% (10-60)	40% (15-60)	30% (10-55)	0.1-0.4%
**Hypercholesteremia**				
Probability of cardiovascular event	10% (5-15)	10% (5-15)	8% (5-10)	2-3%
Estimate of individual benefit with statin therapy	20% (5-50)	25% (10-50)	20% (5-50)	0.3-2%

© 2021 Morgan DJ et al. *JAMA Network Open.*
